# Promoter Hypermethylation-Driven NPHS2 Silencing Promotes Immune Escape and Sunitinib Resistance in Clear Cell Renal Cell Carcinoma

**DOI:** 10.32604/or.2026.080228

**Published:** 2026-09-14

**Authors:** Shangjian Li, Qipeng Han, Xinying Sun, Rongrong Yu

**Affiliations:** 1 Department of Urology, Shandong Provincial Hospital Affiliated to Shandong First Medical University, Jinan, China; 2 Department of Urology, Yiyuan County People’s Hospital, Zibo, China; 3 Department of Oncology, Guangrao County Traditional Chinese Medicine Hospital, Dongying, China

**Keywords:** Nephrosis 2, idiopathic, steroid-resistant, clear cell renal cell carcinoma, immune microcirculation, epigenetic modification, bioinformatics analysis

## Abstract

**Objective:** Renal cell carcinoma is a common malignancy of the urinary system. In this study, we analyzed a public clear cell renal cell carcinoma (ccRCC) dataset and identified Nephrosis 2, idiopathic, steroid-resistant (NPHS2) as a candidate gene to investigate whether epigenetic dysregulation of NPHS2 is associated with tumor microenvironment remodeling. **Methods:** Differential expression analysis was first performed on GSE68417 using GEO2R. In addition, clinical samples and cell-based assays were used to evaluate changes in NPHS2 expression and promoter methylation following 5′-Aza-CdR treatment. Subsequently, 786-O and A498 cells were obtained, and sunitinib-resistant 786-O/R and A498/R sublines were established. Lentiviral vectors with abnormal expression of NPHS2 were transfected into 786-O, A498, 786-O/R, and 498-R cells to detect changes in biological behaviors such as cell activity and epithelial-mesenchymal transition (EMT). Finally, a ccRCC tumor-bearing mouse model was constructed and intervened with lentiviral vectors with abnormal expression of NPHS2. **Results:** A total of 159 differentially expressed genes (DEGs) were identified in the GSE68417 dataset, and NPHS2 was included in all morphological changes related to cell epigenetic modifications. 5′-Aza-CdR markedly increased NPHS2 protein expression in 786-O cells (*p* < 0.05). Meanwhile, there was a high methylation phenomenon in the promoter region of the NPHS2 gene in ccRCC tissue (*p* < 0.05). *In vitro*, NPHS2 overexpression suppressed malignant cell behavior, attenuated EMT, and weakened immune evasion in ccRCC cells (*p* < 0.05). Finally, upregulating NPHS2 could inhibit the growth of ccRCC (*p* < 0.05) without causing significant changes in liver and kidney functions or malignant pathological damage in mice. **Conclusion:** These findings support an association between promoter hypermethylation and NPHS2 downregulation in ccRCC and suggest that NPHS2 loss may contribute to immune-evasive features and reduced sunitinib sensitivity. Further multicohort and mechanistic studies are warranted.

## 1 Introduction

Renal cell carcinoma is a common malignancy of the urinary system, with kidney clear cell renal cell carcinoma (ccRCC) being the most common, accounting for approximately 85% of all kidney cancers [1]. Although the incidence of ccRCC is not prominent compared to tumors in the respiratory and digestive tracts, global epidemiological statistics demonstrate that the global incidence of ccRCC reached approximately 3.8 per 100,000 individuals in 2023, an increase of 16–29% compared with 2010 levels [2]. Meanwhile, due to the typical malignant invasion effect of ccRCC, about 33% of patients are identified with metastatic disease at the time of initial diagnosis and eventually develop metastatic ccRCC, at which point overall survival is often limited to 10–12 months, and the 5-year survival rate will be less than 5% [3]. Tyrosine kinase inhibitors (TKIs, e.g., sunitinib) and immune checkpoint inhibitor therapy represent the first-line standard treatments for ccRCC [4], as this tumor type is intrinsically insensitive to conventional chemotherapy, which is only utilized in a few special clinical scenarios [5]. However, most patients receiving targeted or immunotherapy eventually develop tumor resistance, a major contributor to poor clinical prognosis [6]. At present, studies have confirmed that there is a direct relationship between chemoresistance of tumor cells and changes in the cellular tumor microenvironment (TME) [7], and how to regulate this process has become a crucial target for overcoming therapy resistance (including resistance to tyrosine kinase inhibitors and immune checkpoint blockade) in ccRCC.

Accumulating evidence indicates that epigenetic dysregulation contributes to oncogene activation and tumor suppressor gene silencing in ccRCC [8,9]. In the epigenome analysis of ccRCC, researchers found that almost all ccRCCs have abnormally methylated genes, and the process of methylation modification, in turn, promotes the occurrence and progression of ccRCC [10,11]. Moreover, epigenetic modification is also a major regulatory source affecting various physiological behaviors of cells. For example, epigenetic changes in peripheral T-cell lymphoma can affect the treatment progress [12], and proteomic analysis of methylglyoxal-modified proteins reveals the sensitivity of glycolytic enzymes to dicarbonyl stress [13]. For this reason, recent studies have increasingly examined epigenetic events in ccRCC, with particular interest in genes that may reshape the tumor microenvironment. At present, studies have proposed that the epigenetic regulation of lipid droplet formation is regulated by the oncogene JMJD6-DGAT1 [14], or TET2 inhibiting VHL-defect-driven ccRCC by suppressing HIF signaling [15,16]. Nevertheless, ccRCC progression is unlikely to be explained by the alteration of a single gene, and instead reflects broader regulatory disruption [17,18].

In our pilot study, 159 differentially expressed genes (DEGs) were identified from the ccRCC public dataset GSE68417. Furthermore, in the further Gene Ontology (GO) functional enrichment analysis results, Nephrosis 2, idiopathic, steroid-resistant (NPHS2) has captured our attention as it is included in all morphological changes related to epigenetic modification of cells. By reviewing the relevant literature, we found that NPHS2 contains eight exons and is a novel structural gene discovered by researchers in steroid-resistant nephrotic syndrome in 2000 [19]. Podocin, the encoded protein of NPHS2, is specifically expressed in renal podocytes and is an important membrane protein associated with the slit diaphragm [20], belonging to the stomatin protein family and consisting of 383 amino acid residues [21]. Initial studies revealed that mutations in NPHS2 lead to abnormal slit diaphragm function and cause massive proteinuria [22], serving as a common cause not only for familial steroid-resistant nephrotic syndrome but also for sporadic steroid-resistant nephrotic syndrome [23]. Accordingly, NPHS2 has been investigated in various kidney diseases in recent years. For instance, NPHS2 gene polymorphisms exacerbate renal injury caused by focal segmental glomerulosclerosis with COL4A3 mutations [24], and a new NPHS2 mutation (c.865A > G) has been identified in Chinese families with steroid-resistant nephrotic syndrome [25]. However, in all studies on malignant tumors, including kidney tumors, the role of NPHS2 remains unclear.

Accordingly, this study investigated NPHS2 in ccRCC through integrated bioinformatic, clinical, cellular, and animal analyses, with a focus on its methylation status and its relationship with tumor microenvironment remodeling.

## 2 Materials and Methods

### 2.1 Database Selection

The GSE68417 dataset, generated on the GPL6244 Affymetrix Human Gene 1.0 ST Array platform, was downloaded for analysis. The dataset included high-grade ccRCC samples (Fuhrman grades 3–4 [26], *n* = 16), low-grade ccRCC samples (Fuhrman grades 1–2, *n* = 13), normal kidney tissues (*n* = 14, kidney tissue samples from healthy volunteers), and benign kidney samples (*n* = 6, 2 cases each of renal angiomyolipoma, renal oncocytoma, and renal fibroma), as reported by the original submitter.

### 2.2 DEG Analysis

Differentially expressed genes (DEGs) in GSE68417 were identified using GEO2R (National Center for Biotechnology Information, Bethesda, MD, USA; https://www.ncbi.nlm.nih.gov/geo/geo2r/?acc=GSE68417) [27]. Genes with missing values ≤10%, |logFC| ≥3, and *p* < 0.05 were considered DEGs. The Benjamini–Hochberg false discovery rate (FDR) method was applied for multiple-testing correction to reduce false-positive results. The relatively stringent |logFC| ≥3 threshold was used to identify genes with robust differential expression associated with ccRCC progression, although genes with moderate but potentially relevant biological effects may have been excluded. GO enrichment analysis was performed based on the identified DEGs [28]. GO annotation information was obtained from the org.Hs.eg.db annotation package. All eligible genes detected in GSE68417 after quality filtering were used as the reference background gene set, rather than as mapping targets. Enrichment of GO terms among DEGs was assessed using an over-representation analysis based on the hypergeometric test/Fisher’s exact test. *p*-values were adjusted for multiple comparisons using the Benjamini–Hochberg FDR method, and GO terms with adjusted *p* < 0.05 were considered significantly enriched.

### 2.3 Functional Enrichment Analysis

We obtained the latest gene annotations using the GO annotation of genes in the R software (v 4.4.1, R Foundation for Statistical Computing, Vienna, Austria) package ‘org.Hs.eg.db’ (v. 3.20.0, Bioconductor Project, Seattle, WA, USA) and the Kyoto Encyclopedia of Genes and Genomes (KEGG) REST API (https://www.kegg.jp/kegg/kegg1.html) [29], and used them as background to map genes to the background set.

### 2.4 Protein-Protein Interaction (PPI) Network Construction

We constructed the PPI network by employing the PPI network analysis platform STRING (https://www.string-db.org/, v 12.0, SIB Swiss Institute of Bioinformatics, Lausanne, Switzerland) [30]. The CytoHubba plug-in in Cytoscape (https://cytoscape.org/, v 3.8.2, Cytoscape Consortium, San Diego, USA) was utilized to screen the PPI network model [31]. The core target genes were visually presented through the Maximal Clique Centrality algorithm.

### 2.5 Expression and Prognosis Analysis

The expression of DEGs in Kidney clear cell carcinoma (KIRC) (in online databases, KIRC is commonly used for ccRCC) and other malignant tumor diseases was screened out in the GEPIA database (http://gepia.cancer-pku.cn/index.html, Peking University, Beijing, China) [32], and box expression diagrams were drawn. Additionally, we depicted the prognostic survival differences of the core target genes in different tumor diseases according to their high and low expression levels.

### 2.6 TME Analysis

The CIBERSORT algorithm in the TIMER database (https://cistrome.shinyapps.io/timer/, Harvard T. H. Chan School of Public Health, Boston, USA) was used to estimate the infiltration abundance of immune cells in each sample [33]. The TIMER database was utilized to analyze the relationship between NPHS2 expression and the infiltration of six major immune cells (CD8^+^ T cells, CD4^+^ T cells, B cells, dendritic cells, macrophages, and neutrophils) in the tumor TME [34]. Pearson correlation analysis was used to calculate the quantitative correlation between NPHS2 expression and immune cell infiltration abundance, with the correlation coefficient (R) and statistical significance (P) as the core quantitative indicators for subsequent analysis.

For somatic copy number alteration (SCNA) analysis, the SCNA module of TIMER was used to evaluate whether NPHS2 copy number variation was associated with immune infiltration in ACC, KICH, KIRC, and KIRP. NPHS2 SCNA status was classified according to GISTIC 2.0 into five categories: deep deletion, arm-level deletion, diploid/normal, arm-level gain, and high amplification. The infiltration levels of B cells, CD8^+^ T cells, CD4^+^ T cells, macrophages, neutrophils, and dendritic cells were compared across different NPHS2 SCNA categories. Differences in immune infiltration between each SCNA category and the diploid/normal reference group were assessed using a two-sided Wilcoxon rank-sum test. *p* < 0.05 was considered statistically significant.

### 2.7 Clinical Sample Collection and Processing

A total of 16 ccRCC tissue samples were collected for exploratory analysis of NPHS2 expression and promoter methylation status due to the preliminary nature of this study. Tumor tissues and paired adjacent tissues (>5 cm from the tumor margin) were collected from eight patients with ccRCC who underwent surgery at Shandong Provincial Hospital Affiliated to Shandong First Medical University between January and April 2024. In addition, peripheral blood samples (3 mL) were obtained from all subjects. All specimens were stored at −80°C until analysis. All patients were diagnosed as ccRCC by pathological biopsy, aged ≤80, informed of this study, and signed a consent form. This study involving human subjects complied with the Declaration of Helsinki. The Human Ethics Committees of Shandong Provincial Hospital Affiliated to Shandong First Medical University approved this study (SWYX: NO.2024lun-008). The baseline clinicopathological characteristics of the enrolled patients were as follows: median age 58 years (range 42–76 years); 5 males and 3 females; 2 cases of Fuhrman grade 1, 4 cases of grade 2, 2 cases of grade 3; TNM stage: 3 cases of stage I, 3 cases of stage II, 2 cases of stage III; all patients received no preoperative targeted, immunological or chemotherapeutic treatment.

### 2.8 Western Blot

Total protein was extracted with RIPA buffer (89901, Thermo Fisher Scientific, Waltham, MA, USA), quantified by BCA assay (Thermo Fisher Scientific, Waltham, MA, USA), and subjected to SDS-PAGE (Beyotime Biotechnology, Shanghai, China) using 40 μg protein per lane. After transfer and blocking with 5% skim milk for 2 h, membranes were incubated overnight at 4°C with primary antibodies against NPHS2 (1:1000, ab50339) and GAPDH (1:1000, ab181602), followed by incubation with the secondary antibody (1: 500, ab6721) at room temperature for 2 h. Bands were visualized by ECL (GERPN2232, MilliporeSigma, Billerica, MA, USA) and quantified with ImageJ (v 1.8.0, National Institutes of Health, Bethesda, MD, USA). All antibodies were purchased from Abcam (Cambridge, UK).

### 2.9 Cell Data

786-O and A498 cells, purchased from BeNa Culture Collection (Beijing, China), were immersed in 10% fetal bovine serum (Gibco, Thermo Fisher Scientific, Waltham, USA)—supplemented RPMI 1640 medium (Gibco, Thermo Fisher Scientific, Waltham, USA) and cultured in an incubator set at 37°C and with 5% CO_2_. Passage was carried out when the cell growth length reached 80–90%, once every 3–5 d on average. The cell lines used in this study should be declared as having been identified by Short Tandem Repeat (STR) and be free from mycoplasma contamination. Logarithmic-growth-phase cells were selected for subsequent experiments.

### 2.10 Methylation Analysis

Methylation of gene promoter region: 786-O cells were seeded at a density of 5 × 10^5^ cells/well in 6-well plates and cultured to 70% confluency, then treated with 5′-Aza-CdR (189828, Sigma-Aldrich, Merck KGaA, Darmstadt, Germany), dissolved in dimethyl sulfoxide (DMSO, D2650; Sigma-Aldrich, Merck KGaA, Darmstadt, Germany) at concentrations of 5 and 10 μmol/L for 24 h with no medium change during treatment, and then NPHS2 protein expression was detected. Tissue genomic DNA was extracted using a kit (69504, Qiagen, Hilden, Germany), and the genomic DNA was transformed and purified following the Bisulfite Conversion Kit instructions (585459, Qiagen, Hilden, Germany). The NPHS2 promoter region (about −350~−200 bp upstream of the transcription initiation site) contains a methylation island. The online software Meth Primer 2.0 (http://www.urogene.org/cgi-bin/methprimer/methprimer.cgi1, University of California, San Francisco, CA, USA) was used to design methylated primer pairs and non-methylated primer pairs for MSP (Table 1). The MSP reaction system (25 μL) contained 12.5 μL 2× Taq PCR Master Mix, 1 μL each of forward and reverse primers (10 μmol/L), 2 μL bisulfite-modified DNA template, and 8.5 μL ddH_2_O. Using the transformed DNA as a template, PCR amplification was performed with Taq DNA polymerase. The PCR cycling parameters were as follows: pre-denaturation at 95°C for 5 min; 95°C for 30 s, 72°C for 30 s; 72°C for 10 min. Methylated human genomic DNA served as the positive control, and ddH_2_O as the negative control. Then, the methylated and unmethylated amplification products were electrophoresed in a 2% agarose gel, and the DNA bands were observed and photographed.

**Table 1:** Primer information for MSP of the NPHS2 promoter region.

Primer Type	Sequence (5′–3′)	Annealing Temperature (°C)	Amplification Product Length (bp)
Methylated forward	GTTTTAGAGCGGTCGGTATTTC	58	187
Methylated reverse	ACGACCTACGACGAAACGTA	58	/
Unmethylated forward	GTTTTAGAGTGGTTGGTATTTT	56	195
Unmethylated reverse	ACAACCTACAACGAAACATA	56	/

### 2.11 Construction of Drug-Resistant Cells

To optimize the growth state and drug resistance screening efficiency of ccRCC cells, the culture medium was switched from RPMI 1640 to high-glucose DMEM (Gibco, Thermo Fisher Scientific, Waltham, USA), and the parental 786-O and A498 cells (control group) were also cultured in the same DMEM medium to eliminate confounding effects caused by medium differences. Sunitinib-resistant sublines were established by stepwise exposure to sunitinib at 2.5, 7.5, 12.5, and 20.0 μmol/L (SU11248, TargetMol Chemicals Inc., Boston, MA, USA). Cells were passaged after stable growth at each concentration, and resistant lines were generated after approximately 8 weeks, designated 786-O/R and A498/R. Cell growth curves were then evaluated by CCK-8 assay.

### 2.12 Lentivirus Packaging

When cell confluence reached 30%–40%, 786-O, A498, 786-O/R, and A498/R cells were infected with NPHS2 overexpression or control lentivirus at a multiplicity of infection of 20 in the presence of 2.5 μg/mL polybrene (11642, Sigma-Aldrich, Merck KGaA, Darmstadt, Germany). Puromycin selection (2–5 μg/mL, Sigma-Aldrich, Merck KGaA, Darmstadt, Germany) was initiated 72 h later, and stable cells were collected for subsequent assays. 786-O and A498 cells transfected with the NPHS2 overexpression vector and the empty vector were labeled as NPHS2-ov and NPHS2-nc, respectively; 786-O/R and A498/R cells intervened by the NPHS2 overexpression vector and the empty vector were labeled as NPHS2-ov/R and NPHS2-nc/R, respectively. The lentiviral vectors contained a green fluorescent protein reporter to monitor transfection efficiency. At 72 h after infection, green fluorescence was observed under an inverted fluorescence microscope. Transfection efficiency was evaluated by calculating the percentage of GFP-positive cells among total cells in randomly selected fields. Cells with stable fluorescence expression after puromycin selection were used for subsequent experiments. NPHS2 overexpression efficiency was further confirmed by Western blot.

### 2.13 Determination of Sunitinib IC50 and Resistance Index

The sensitivity of parental and sunitinib-resistant ccRCC cells to sunitinib was evaluated using the CCK-8 assay. Briefly, 786-O, A498, 786-O/R, and A498/R cells in the logarithmic growth phase were seeded into 96-well plates at a density of 1× 10^3^ cells/well and cultured overnight. Cells were then treated with different concentrations of sunitinib for 48 h. Subsequently, 10 μL of CCK-8 reagent was added to each well and incubated at 37°C for 2 h. The absorbance at 450 nm was measured using a microplate reader. Cell viability was calculated relative to the untreated control group, and the half-maximal inhibitory concentration (IC50) was determined by nonlinear regression analysis based on the dose–response curve.

### 2.14 Cell Activity Detection

#### 2.14.1 EdU

786-O and A498 seeded in a 24-well plate (1 × 10^5^/mL) were added with EdU solution (50 μmol/L) and incubated for 2 h according to the instructions of the EdU Cell Proliferation Assay Kit (C0088S, Beyotime Biotechnology, Shanghai, China), and fixed with 4% paraformaldehyde. They were then stained with Hoechst and Apollo solutions in the kit and photographed under the inverted fluorescence microscope (EVOS^TM^ M3000 Imaging System, Thermo Fisher Scientific, Waltham, MA, USA). Cells under different fluorescence conditions were counted using ImageJ’s automated cell counting function, and the cell proliferation rate was recorded.

#### 2.14.2 Cell Cloning

786-O and A498 were inoculated into a 6-well culture plate (3 × 10^2^ per well), with 3 replicate wells in each group, followed by routine culture in a DMEM medium containing 10% fetal bovine serum, with the medium replaced every 2–3 days. When visible cell clones were observed (about 10 ds), the culture medium was discarded, and the clones were fixed with 4% paraformaldehyde (30 min), dried, and stained with 0.1% crystal violet (20 min). Colonies were observed after PBS (pH 7.4, 1× PBS) washing, and those with more than 50 cells were counted.

#### 2.14.3 Cell Invasion

786-O and A498 were added to Transwell inserts (Corning Incorporated, Corning, NY, USA) in quantities of 30,000 per well. 500 μL of RPMI-1640 containing 10% FBS was added to the lower layer of the chamber, and the FBS concentration of the culture medium in the chamber was 1%. Next, the chamber was incubated in a 37°C, 5% CO_2_ incubator for 24 h. Then came 4% paraformaldehyde fixation for 10 min, PBS (pH 7.4, 1× PBS) wash 3 times, 0.1% crystal violet dye staining for 30 min, and PBS rinse 3 times. Finally, following the removal of unmigrated tumor cells in the chamber with moistened cotton swabs, photographed under a microscope (CX23, Olympus Co., Ltd., Shanghai Branch, Shanghai, China), and the total number of cells passing through the chamber was counted.

#### 2.14.4 Cell Scratch Assay

When the 786-O and A498 cells inoculated into the 6-well plate (4 × 10^5^/well) grew to be adherent and confluent, a 200 μL pipette tip was used to scrape off the intermediate cells, followed by culture for 24 h. Cell mobility = (0 h width − 24 h width)/0 h width × 100%.

#### 2.14.5 Cell Cycle and Apoptosis

For cell cycle detection, the collected 786-O and A498 were fixed with 70% cold ethanol at 4°C overnight, then washed with PBS (pH 7.4, 1× PBS) and incubated with PI/RNase Staining Buffer (50 μg/mL PI, 100 μg/mL RNase A, HY000525, Shanghai Yes Service Biotech, Inc., Shanghai, China) at room temperature for 30 min in the dark. Cell cycle distribution was analyzed by flow cytometry (Attun^TM^ NxT flow cytometer, Thermo Fisher Scientific, Waltham, MA, USA), and the percentage of cells in G0/G1, S, and G2/M phases was calculated. Changes in cell cycle were determined by flow cytometry, and levels of apoptosis-related proteins Bax (1:1000, ab32503), Bcl-2 (1:1000, ab182858), Caspase-3 (1:1000, ab184787), and cleaved Caspase-3 (1:1000, ab32042) in cells were quantified. The methods refer to the “Western blot” section previously (Section 2.8).

### 2.15 Cell Biological Behavior Assays

#### 2.15.1 Immune Escape

For CD8, PD-1, and PD-L1 detection, 786-O and A498 were co-cultured with human peripheral blood mononuclear cells (PBMCs, isolated from healthy donor peripheral blood by Ficoll density gradient centrifugation [35]) at a ratio of 1:10 in RPMI 1640 medium containing 10% FBS for 48 h, and CD8 was detected on the surface of T cells in the co-culture system. After blocking for 30 min at room temperature in the dark using 5% BSA bovine serum albumin, CD8 (1:500, ab237709), PD-1 (1:500, ab52587), and PD-L1 (1:500, ab205921) primary antibody (Abcam, Cambridge, UK) solutions were added for overnight incubation in the dark before washing. After that, a second antibody (1:2000, ab120493) was added to incubate, and then a DAPI staining solution (1 μg/mL, 10 min) was added. Following rinsing and mounting, the staining results were observed under a fluorescent microscope (EVOS^TM^ M3000 Imaging System, Thermo Fisher Scientific, Waltham, MA, USA).

#### 2.15.2 Mitochondrial Damage

786-O and A498 cells were seeded in 24-well plates at a density of 4 × 10^4^ cells per well and continued to be cultured in an incubator for 48 h. Subsequently, the cell culture solution was removed, and the prepared JC-1 staining working solution (M34152, Thermo Fisher Scientific, Waltham, MA, USA) was added at 250 μL per well, for 20 min of culture at 37°C in the dark. After two washes with 4°C pre-chilled JC-1 staining buffer (1×), the photos were taken under the fluorescence microscope (red fluorescence occurs when the mitochondrial membrane potential is high, and green fluorescence occurs when it decreases).

#### 2.15.3 Oxidative Stress Injury

The supernatant of 786-O and A498 cultures was collected for the determination of superoxide dismutase (SOD) (CSB-RA022397MA1HU), malondialdehyde (MDA) (CSB-E15840h), and glutathione peroxidase (GSH-Px) (CSB-E13068h) by ELISA kits (Wuhan Huamei BioEngineering Co., Ltd., Wuhan, Hubei, China), with protein concentration (detected by BCA) as the normalization standard. The intracellular reactive oxygen species (ROS) fluorescence intensity was detected by DCFH-DA staining (RC08155-100T, Shanghai Lusen Bioengineering Co., Ltd., Shanghai, China) according to the above method, and the fluorescence intensity was normalized to the number of viable cells.

#### 2.15.4 Epithelial-Mesenchymal Transition (EMT)

Western blot was used to detect E-Cadherin (1:1000, ab40772), N-Cadherin (1:1000, ab76011), and Vimentin (1:1000, ab92547) protein levels in cells, using the same method as above. Methods refer to the “Western blot” section above (Section 2.8).

#### 2.15.5 T Cell Activation

786-O and A498 in logarithmic growth phase were taken and digested with 0.25% trypsin—0.53 mM EDTA (Gibco, Thermo Fisher Scientific, Waltham, USA), and the concentration was adjusted to 1 × 10^6^/mL. Flow cytometry was used to detect CD69^+^ (antibody: CD69-PE, MA5-16687), PD-1^+^ (antibody: PD-1-APC, PA5-122120), TIM-3^+^ (antibody: TIM-3-FITC, MA5-32839), IFN-γ (antibody: IFN-γ-PerCP-Cy5.5) (61-7311-82) (Thermo Fisher Scientific, Waltham, MA, USA). A total of 100 μL of the cell suspension was used, and the surface antibody was added. 2% Fixative solution (BDB554655, BD Cytofix Buffer, Shanghai Yes Service Biotech, Inc., Shanghai, China) was added and incubated for 20 min at 4°C. The supernatant was discarded by centrifugation and washed once with membrane-breaking solution (BDB516426, BD Permeabilization Wash Buffer, Shanghai Yes Service Biotech, Inc., Shanghai, China). Intracellular antibody was added, washed twice with membrane-breaking solution, and resuspended in PBS (pH 7.4, 1× PBS) before detection.

### 2.16 Animal Data

Twenty 4-week-old SPF-grade healthy C57BL/6 nude mice, weighing 18–20 g, were purchased from Cyagen Biosciences (Suzhou, China) Inc. [SCXK(Su)2022-0016]. All animal procedures were approved by the Institutional Animal Care and Use Committee of Shandong Provincial Hospital Affiliated to Shandong First Medical [approval No. SWYX: NO. 2021-308] and were conducted in accordance with institutional guidelines and the ARRIVE 2.0 essential 10 recommendations [36]. Mice were acclimatized for 7 days before experiments and housed under SPF conditions, with five mice per cage, at 22–25°C and 40%–60% humidity under a 12-h light/dark cycle, with free access to food and water. C57BL/6 nude mice are T-cell-deficient and were used to establish subcutaneous tumor models; tumor-infiltrating lymphocytes were mainly derived from residual innate immune cells and immune cells recruited into tumor tissues. Mice were randomly allocated to experimental groups, and investigators were blinded during outcome assessment where feasible. Body weight, tumor growth, and general health were monitored regularly. Humane endpoints included excessive tumor burden, ulceration, marked weight loss, impaired mobility, or severe distress.

### 2.17 Construction of ccRCC Tumor-Bearing Mice

Twenty mice were randomly assigned to the NPHS2-ov and NPHS2-nc groups (*n* = 10 each). 786-O cells (5 × 10^6^ in 500 μL) were injected subcutaneously into the left axilla to establish xenografts. Beginning on d 7 after inoculation, mice received tail-vein injection of lentivirus (1 × 10^8^ TU/mL, 100 μL/d) for 7 consecutive ds (Changsha ABIVay Biotech Co., Ltd., Changsha, Hunan, China). Mice were euthanized 14 ds after the first injection, and tumors were harvested for measurement and subsequent analyses.

### 2.18 Tumor Volume Detection

As mentioned previously, all mice were sacrificed after 14 days. The complete tumor tissue was removed, and the long and short diameters of the tumor were measured with vernier calipers to calculate the volume (V) = 1/2 × major diameter × minor diameter^2^.

### 2.19 Immunohistochemistry (IHC)

Mouse tumor tissue was sectioned, treated with antigen retrieval solution, and added with an NPHS2 antibody (1:100, ab50339, Abcam, Cambridge, UK) at 4°C overnight. The sections were processed with reagents from the Eli Vision Super kit (N313-KT, Amresco, Brunswick, OH, USA) at room temperature for 1 h, followed by color development in 0.05% diaminobenzidine (DAB) (D12384, Sigma-Aldrich, Merck KGaA, Darmstadt, Germany) and the subsequent observation and photographing microscopically. The IHC staining results were scored based on both staining intensity and positive cell proportion. Dyeing depth: no staining, light staining, moderate to strong staining, and strong staining were recorded as 0, 1, 2, and 3, respectively. Positive cell proportion: <10%, 10%~<25%, 25%~<50%, 50%~<75%, and ≥75% were assigned a score of 0, 1, 2, 3, and 4 points, respectively. The total IHC staining score = the staining intensity score × positive cell proportion score.

### 2.20 HE Staining

At the end of the experiment, mice were euthanized according to the approved animal protocol. Liver and kidney tissues were carefully isolated, rinsed with cold phosphate-buffered saline to remove residual blood, and fixed in 4% paraformaldehyde for 24–48 h. The fixed tissues were dehydrated through graded ethanol, cleared in xylene, embedded in paraffin, and sectioned at a thickness of 4–5 μm. The sections were then stained with hematoxylin and eosin according to standard procedures. Histopathological changes, including inflammatory cell infiltration, tissue necrosis, congestion, cellular swelling, and structural damage, were observed under a light microscope by investigators blinded to group allocation.

### 2.21 Biochemical Tests

Before euthanasia, blood samples were collected from mice by abdominal aorta puncture. After standing at room temperature for 30 min, blood samples were centrifuged at 3000 rpm for 10 min to obtain serum. Serum liver and kidney function indicators, including alanine aminotransferase, aspartate aminotransferase, blood urea nitrogen, and serum creatinine, were measured using an automatic biochemical analyzer (BS-600M, Mindray Medical International Co., Ltd., Shenzhen, Guangdong, China) according to the manufacturer’s instructions.

### 2.22 Flow Cytometry Analysis

Mouse subcutaneous tumor tissues were removed under sterile conditions, washed with precooled PBS (containing 2% FBS), and digested with 0.25% trypsin to prepare a tumor tissue single-cell suspension. Viable cells were counted by trypan blue staining (T8154, Sigma-Aldrich, Merck KGaA, Darmstadt, Germany), and the concentration was adjusted to 1 × 10^7^/mL. Subsequently, flow cytometry was used to detect CD8^+^ T cells (antibody: CD8a-FITC) (PSI-76-684, ProSci Incorporated, San Diego, CA, USA), CD4^+^ Th1 cells (antibody: CD4-APC, IFN-γ-PE-Cy7) (IM2468, Beckman Coulter, Brea, CA, USA), Treg cells (antibody: CD4-APC, CD25-PE, Foxp3-PerCP-Cy5.5) (PCP55-30055, Wuhan Fine Biological Technology Co., Ltd., Wuhan, Hubei, China). Same method as above.

### 2.23 Statistical Methods

Statistical analyses were performed using SPSS 24.0 (IBM, Armonk, NY, USA). Data distribution was assessed using the Shapiro–Wilk test, and homogeneity of variances was evaluated using Levene’s test. Normally distributed data are presented as mean ± standard deviation. For comparisons between two groups, the independent-samples *t* test was used when variances were homogeneous, whereas Welch’s *t* test was applied when variances were unequal. For comparisons among multiple groups, one-way ANOVA was performed when normality and homogeneity of variance assumptions were met. LSD post hoc analysis was used only for preplanned pairwise comparisons when the overall ANOVA was significant, and the number of comparisons was limited; otherwise, a more conservative post hoc test, such as Bonferroni or Games–Howell, was applied as appropriate. Non-normally distributed data are presented as median (interquartile range) and were analyzed using the Mann–Whitney U test or Kruskal–Wallis test, as appropriate. A two-sided *p* < 0.05 was considered statistically significant.

## 3 Results

### 3.1 Bioinformatics Analysis Results

**Figure 1:**
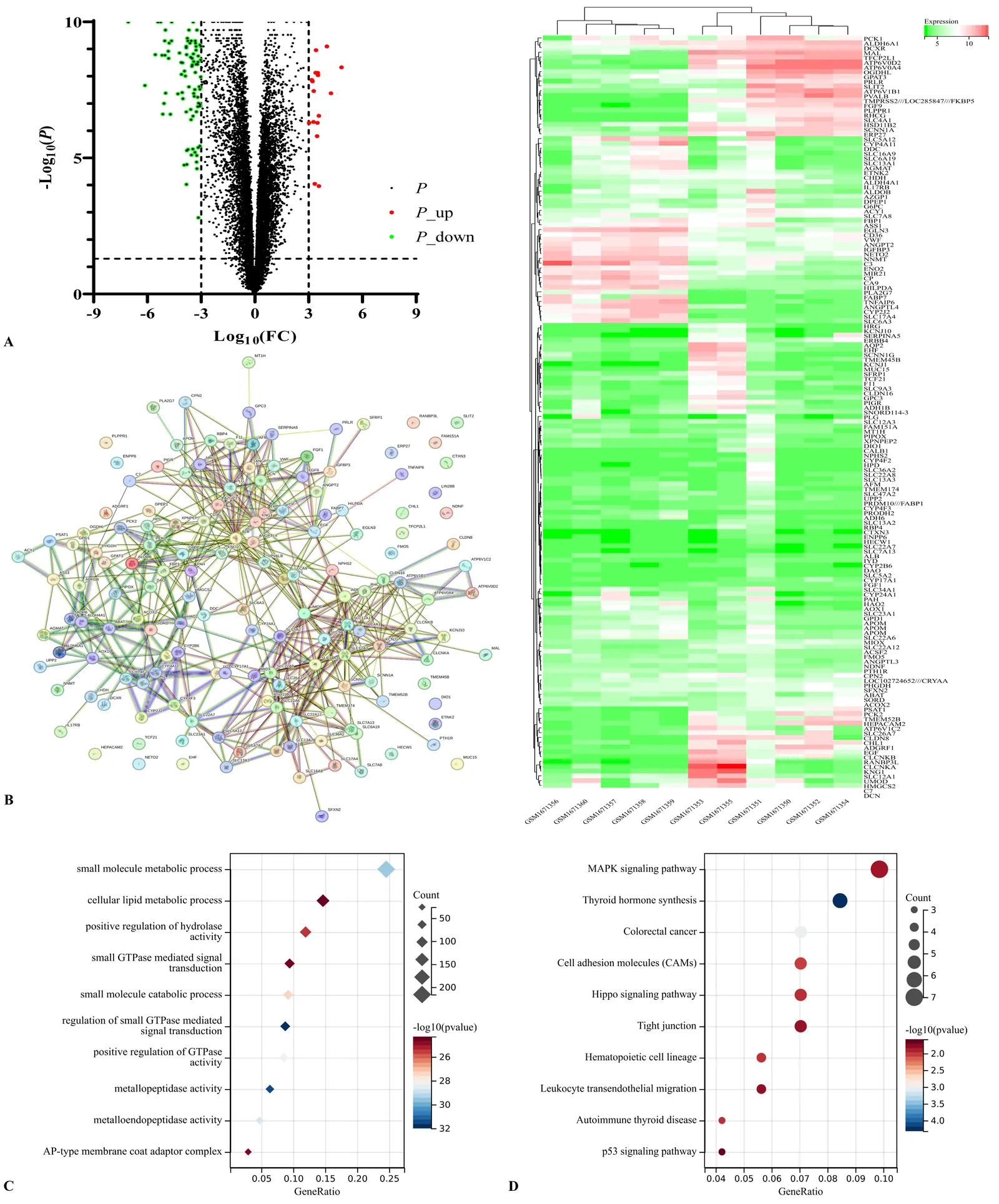
**Bioinformatics analysis results.** (**A**) Volcano map of DEGs with expression heat map. (**B**) PPI network of DEGs. (**C**) GO analysis results of DEGs. (**D**) KEGG analysis results of DEGs.

After removing all unnamed and duplicate genes, we screened out 159 DEGs from GSE68417, among which 20 were upregulated, and 139 were downregulated (Fig. 1A). PPI analysis revealed that these 159 DEGs contained 153 nodes and 517 edges (Fig. 1B). For the functional enrichment analysis, GO showed that the keywords involved in these DEGs contained small molecule metabolic process, cellular lipid metabolic process, and so on (Fig. 1C). And the most strongly associated pathway of the MAPK signaling pathway was seen in KEGG (Fig. 1D). Notably, among these DEGs, NPHS2 was enriched in multiple GO terms specifically associated with kidney development and glomerular epithelial cell differentiation (Table 2), processes known to be susceptible to epigenetic dysregulation in cancer. Therefore, we made it a focus of our subsequent study.

**Table 2:** Functional enrichment results of NPHS2.

GO Code	Functionality	*p*
GO:0031982	Vesicle	<0.001
GO:0070062	extracellular exosome	<0.001
GO:1903561	extracellular vesicle	<0.001
GO:0043230	extracellular organelle	<0.001
GO:0044421	extracellular region part	<0.001
GO:0005576	extracellular region	<0.001
GO:0005615	extracellular space	<0.001
GO:0007588	Excretion	<0.001
GO:0012505	endomembrane system	<0.001
GO:0030036	actin cytoskeleton organization	<0.001
GO:0098805	whole membrane	<0.001
GO:0044459	plasma membrane part	<0.001
GO:0030029	actin filament-based process	<0.001
GO:0030054	cell junction	<0.001
GO:0001822	kidney development	<0.001
GO:0048731	system development	<0.001
GO:0072001	renal system development	0.001
GO:0001655	urogenital system development	0.001
GO:0005911	cell-cell junction	0.002
GO:0007010	cytoskeleton organization	0.001
GO:0072243	metanephric nephron epithelium development	0.005
GO:0045121	membrane raft	0.012
GO:0098857	membrane microdomain	0.012
GO:0072244	metanephric glomerular epithelium development	0.007
GO:0072248	metanephric glomerular visceral epithelial cell differentiation	0.007
GO:0072249	metanephric glomerular visceral epithelial cell development	0.007
GO:0072312	metanephric glomerular epithelial cell differentiation	0.007
GO:0072313	metanephric glomerular epithelial cell development	0.007
GO:0098589	membrane region	0.018
GO:0005783	endoplasmic reticulum	0.018
GO:0072207	metanephric epithelium development	0.009

### 3.2 Verification of Clinical Expression of NPHS2

**Figure 2:**
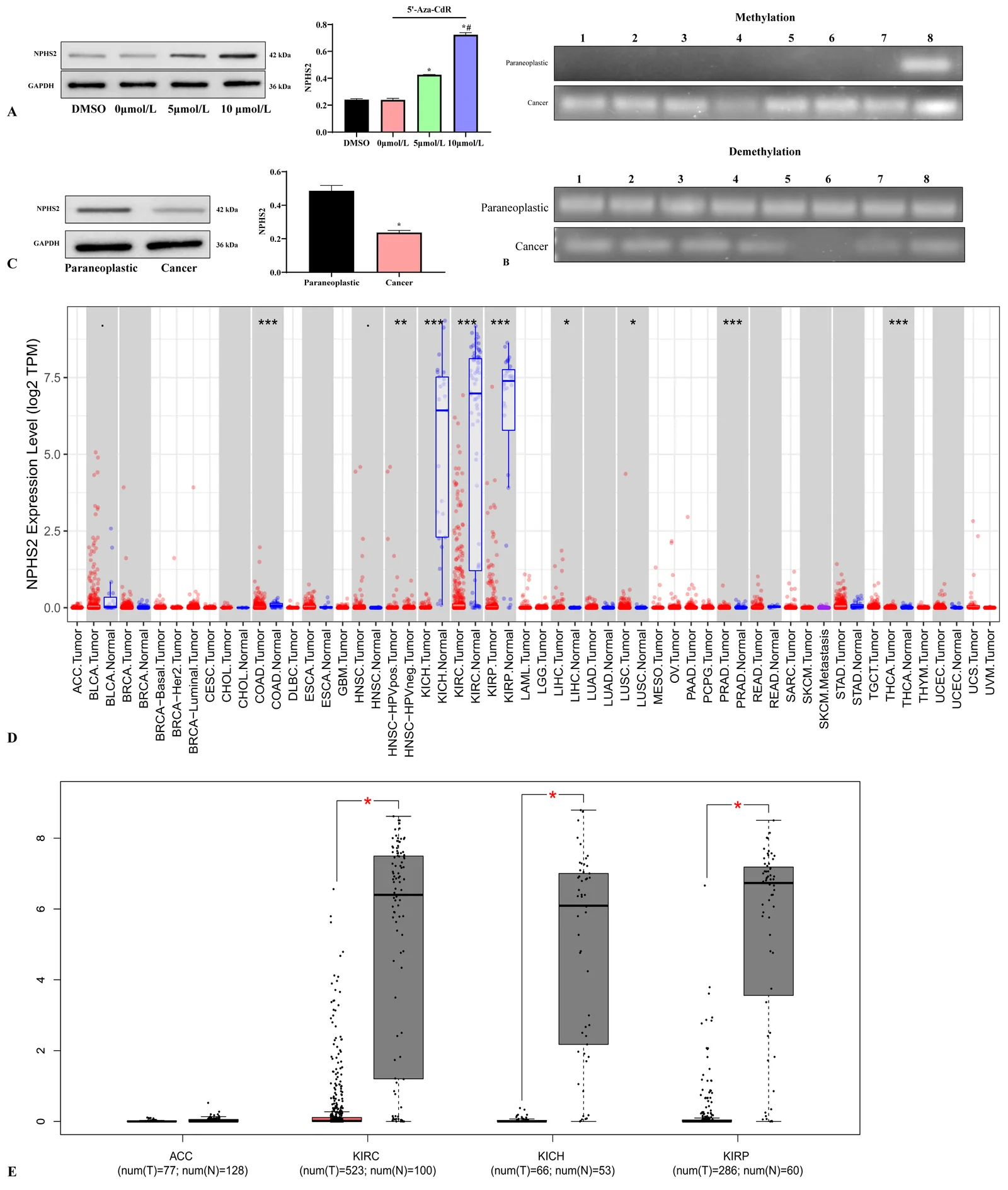
**Verification of clinical expression of NPHS2.** (**A**) Effect of 5′-Aza-CdR on NPHS2 expression. vs. 0 μmol/L, *p < 0.05; vs. 5 μmol/L, ^#^*p* < 0.05; (*n* = 3). (**B**) MSP analysis of NPHS2 promoter methylation in ccRCC and paired adjacent tissues (*n* = 8). (**C**) NPHS2 expression in clinical samples, vs. paraneoplastic tissue *p < 0.05 (*n* = 8). (**D**,**E**) NPHS2 expression in the database. *p < 0.05, **p < 0.01, ***p < 0.001.

Second, the NPHS2 protein level in 5′-Aza-CdR-treated 786-O was notably increased compared with the control group (*p* < 0.05, Fig. 2A), indicating the possible presence of abnormal methylation modifications in 786-O. We further analyzed the MSP method to analyze the methylation level of the NPHS2 gene promoter region in ccRCC and the corresponding adjacent tissues. MSP analysis showed that methylated bands were detected in all ccRCC tissues, whereas only one paired adjacent tissue sample showed a methylated band. In contrast, unmethylated bands were detected in all adjacent tissues and were absent or weaker in parts of the ccRCC tissues. These results suggest that the NPHS2 promoter region was hypermethylated in ccRCC tissues compared with adjacent tissues (Fig. 2B). The MSP detection results indicated that compared with adjacent tissues, the NPHS2 gene promoter region in ccRCC tissues was hypermethylated. According to the GEO2R analysis, NPHS2 was in a downregulated state in ccRCC. For this, we included clinical samples for verification and found statistically reduced NPHS2 protein expression in cancer tissues of ccRCC patients versus adjacent tissues (*p* < 0.05, Fig. 2C), which is consistent with the results of GEO2R analysis. However, the difference in NPHS2 expression between cancer and adjacent tissues showed an opposite trend to its methylation degree. In the GEPIA database, NPHS2 exhibits abnormal expression, specifically down-regulated, only in kidney chromophobe (KICH), KIRC, and kidney renal papillary cell carcinoma (KIRP) (Fig. 2D,E), prompting us to investigate the potential role and regulatory mechanism of NPHS2 in ccRCC. This suggests that the regulation of NPHS2 expression involves epigenetic mechanisms, and its low expression in ccRCC tissue may be related to the hypermethylation of the gene promoter region. These results are based on exploratory analysis with a small sample size (*n* = 8), and the statistical conclusions need to be further verified by large-sample clinical cohort studies.

### 3.3 Analysis of the Influence of NPHS2 on the Tumor TME of ccRCC

**Figure 3:**
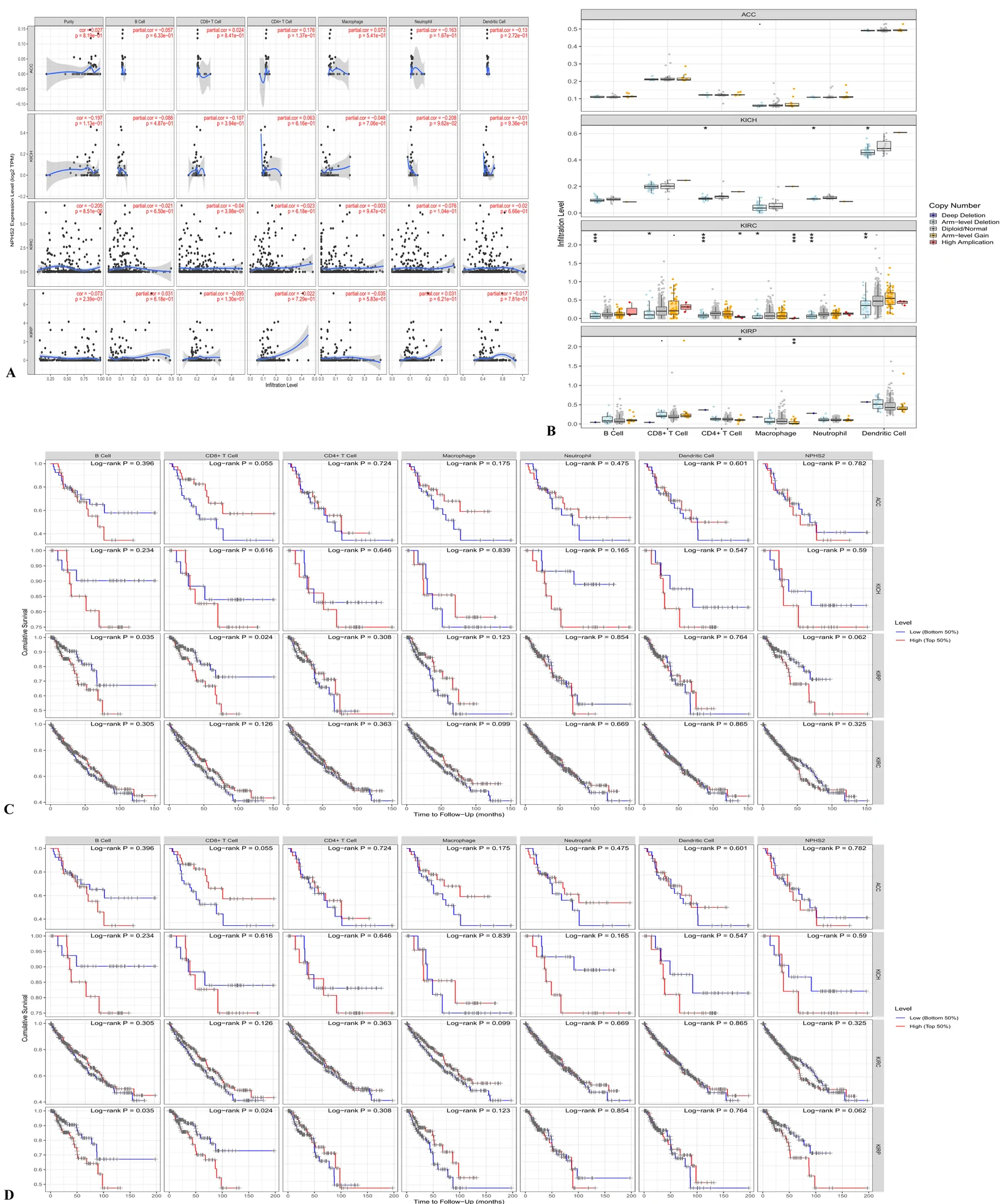
**Analysis of the influence of NPHS2 on the tumor TME of ccRCC.** (**A**) Correlation between NPHS2 and immune infiltrating cells. (**B**) Changes in immune-infiltrating cells at different NPHS2 copy fractions. (**C**) Relationship between NPHS2 and prognosis. (**D**) Relationship between NPHS2 and prognosis after removing confounding factors such as age, sex, and TNM stage. *p < 0.05, **p < 0.01, ***p < 0.001.

TME is the focus of modern tumor research and the key to achieving tumor immunotherapy [37]. On the one hand, immune cells can play an immunosurveillance role in adopting an anti-tumor phenotype, preventing tumor progression; on the other hand, immune cells, under the influence of TME, can adopt a pro-tumor phenotype, permitting tumor escape, or even supporting TME, thus promoting tumor progression. In the TIMER database, we found no significant correlation between NPHS2 and immune cells of KICH, KIRC, KIRP, and adrenocortical carcinoma (ACC) (*p* > 0.05, Fig. 3A). In SCNA analysis, no changes were observed in the immune subgroups of ACC at different NPHS2 copy number variations (*p* > 0.05); yet, significant alterations were found in KICH, KIRC, and KIRP (*p* < 0.05), with KIRC being the most significant (B cells, CD8^+^, CD4^+^, macrophages, neutrophils, and dendritic cells showed statistically significant differences in changes) (Fig. 3B). Meanwhile, in the state of immune infiltration, there was no significant correlation between NPHS2 and the prognosis of KICH, KIRC, and ACC (*p* > 0.05). However, in KIRP, we found that with immune infiltration of B cells and CD8^+^ cells, the prognosis and survival of patients with high NPHS2 expression reduced (*p* < 0.05, Fig. 3C); this result also held under the influence of covariates such as age and sex (Fig. 3D), which is inconsistent with the inspection results of GEO2R mentioned above and requires further verification and discussion.

### 3.4 The Effect of NPHS2 on ccRCC Cell Activity

**Figure 4:**
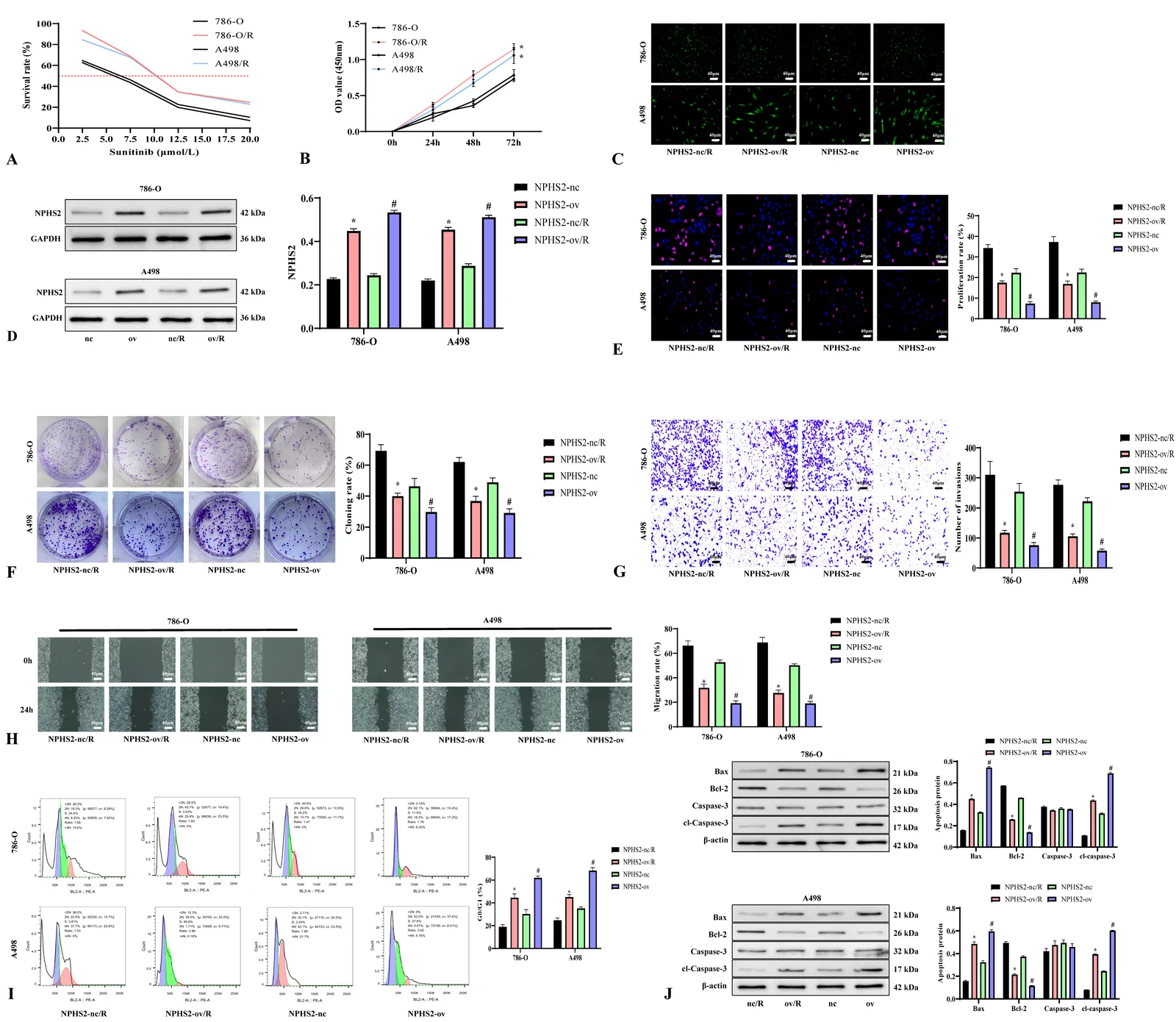
**The effect of NPHS2 on ccRCC cell activity.** (**A**) IC_50_ for sunitinib in resistant and normal cells. (**B**) Growth curves of 786-O, A498, 786-O/R, and A498/R (*n* = 3). (**C**) Representative fluorescence images showing GFP-positive cells after lentiviral infection in 786-O, A498, 786-O/R, and A498/R cells (*n* = 3). (**D**) Validation of the effect of intervention on NPHS2 expression (*n* = 3). (**E**) EdU detection of the effect of NPHS2 on the proliferative capacity of 786-O, A498, 786-O/R, and A498/R (*n* = 3). (**F**) Cell cloning assay to detect the effect of NPHS2 on the cloning ability of 786-O, A498, 786-O/R, and A498/R (*n* = 3). (**G**) Transwell assay to detect the effect of NPHS2 on 786-O, A498, 786-O/R, and A498/R invasion ability (*n* = 3). (**H**) Cell scratch assay to detect the effect of NPHS2 on the migration ability of 786-O, A498, 786-O/R, and A498/R (*n* = 3). (**I**) Effect of flow cytometry on NPHS2 in 786-O, A498, 786-O/R, and A498/R cell cycle. (**J**) Expression of apoptosis-related proteins (*n* = 3). vs. NPHS2-nc group, *p < 0.05; vs. NPHS2-nc-R group, ^#^*p* < 0.05.

Firstly, we could observe that the IC_50_ of both 786-O/R and A498/R was higher than that of normal 786-O and A498 (Fig. 4A). The IC_50_ values of sunitinib for 786-O, 786-O/R, A498 and A498/R were 7.25 ± 0.41 μmol/L, 11.67 ± 2.15 μmol/L, 6.98 ± 0.35 μmol/L and 11.34 ± 1.92 μmol/L, respectively. CCK-8 assays showed that the cell growth trend of 786-O/R and A498/R was also significantly elevated (Fig. 4B), confirming the success of drug-resistant cell construction. After lentiviral infection and puromycin selection, obvious GFP fluorescence was observed in 786-O, A498, 786-O/R, and A498/R cells, indicating successful lentiviral transfection/infection (Fig. 4C). Western blot further confirmed that NPHS2 protein expression was increased in the NPHS2-ov and NPHS2-ov/R groups compared with the corresponding control groups (*p* < 0.05, Fig. 4D). In terms of cell activity, the NPHS2-ov and NPHS2-ov/R groups presented weaker proliferation, cloning, and migration capacities as well as a lower cell invasion number than the NPHS2-nc and NPHS2-nc/R groups (*p* < 0.05, Fig. 4E–H); moreover, the results of cell cycle analysis via PI/RNase staining revealed obviously prolonged G0/G1 phase of cells in the NPHS2-ov and NPHS2-ov/R groups (*p* < 0.05, Fig. 4I), and western blot showed increased levels of pro-apoptosis proteins Bax and cl-caspase-3, and decreased expression of the anti-apoptosis protein Bcl-2 (*p* < 0.05, Fig. 4J). These findings indicate that NPHS2 overexpression restrains the growth of both parental and resistant ccRCC cells while enhancing apoptosis.

### 3.5 Impacts of NPHS2 on ccRCC Cell Biological Behavior

**Figure 5:**
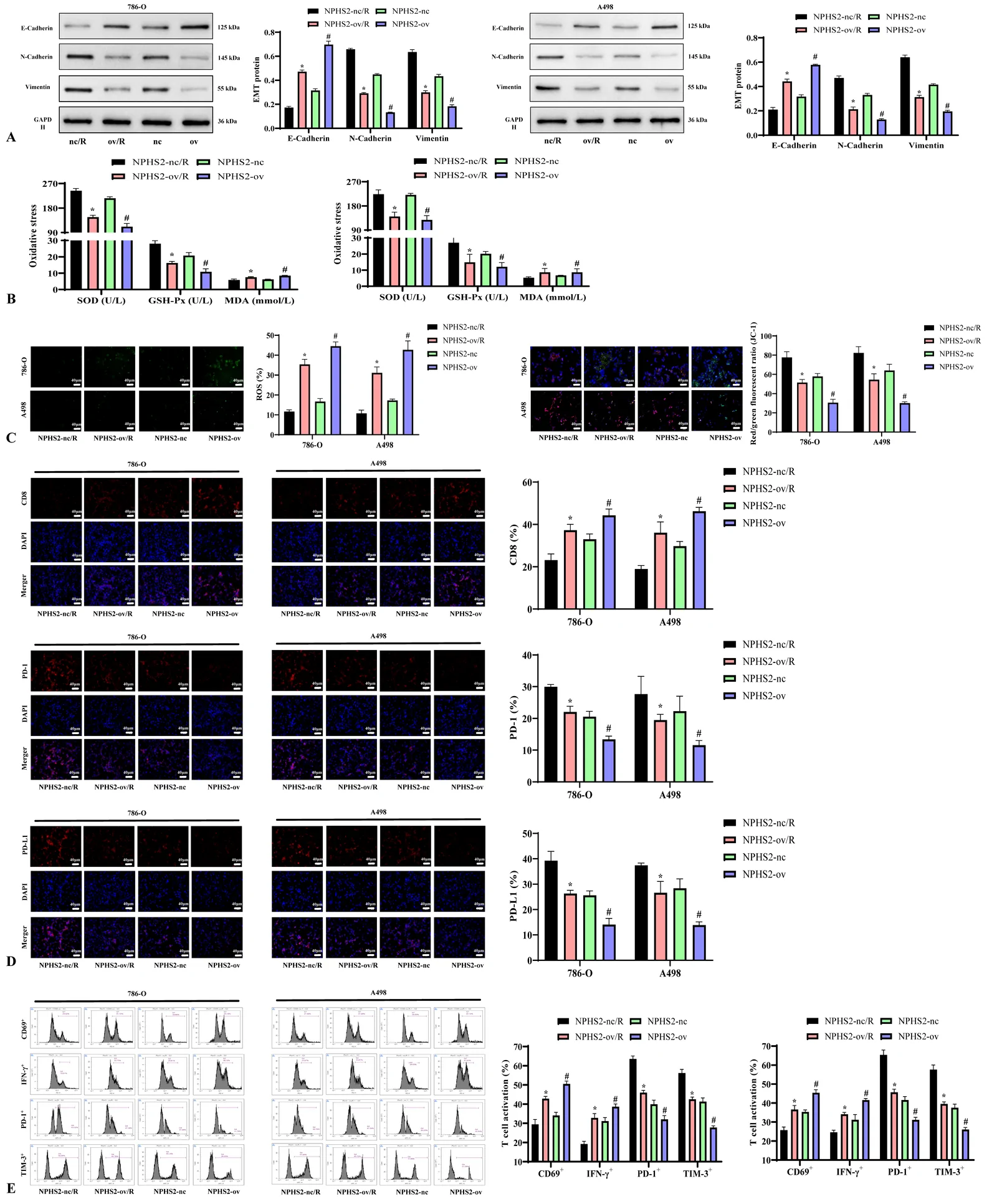
**Impacts of NPHS2 on ccRCC cell biological behavior.** (**A**) Effect of NPHS2 on EMT in 786-O, A498, 786-O/R, and A498/R cells (*n* = 3). (**B**) Effect of NPHS2 on oxidative stress response in 786-O, A498, 786-O/R, and A498/R (*n* = 3). (**C**) Effect of NPHS2 on ROS and mitochondrial damage in 786-O, A498, 786-O/R, and A498/R (*n* = 3). (**D**) Effect of NPHS2 on CD8, PD-1, PD-L1 in 786-O, A498, 786-O/R, and A498/R (*n* = 3). (**E**) Effect of NPHS2 on T cell activation status (CD69^+^, IFN-γ^+^, PD-1^+^, TIM-3^+^) in 786-O, A498, 786-O/R and A498/R. vs. NPHS2-nc group, *p < 0.05; vs. NPHS2-nc-R group, ^#^*p* < 0.05.

Further observation was conducted on the effect of NPHS2 on the biological behavior of ccRCC cells. First of all, in terms of EMT, E-Cadherin protein expression was increased in the NPHS2-ov and NPHS2-ov/R groups, while N-Cadherin and Vimentin protein expression was decreased (*p* < 0.05), confirming that the EMT progress in cells is obviously reversed (Fig. 5A). Regarding oxidative stress damage, the levels of SOD and GSH-Px in the cell supernatant decreased in the NPHS2-ov and NPHS2-ov/R groups, while MDA increased (*p* < 0.05, Fig. 5B), with all indicators normalized to protein concentration. Meanwhile, in cell fluorescence staining, we observed stronger ROS fluorescence intensities in the NPHS2-ov and NPHS2-ov/R groups than in the NPHS2-nc and NPHS2-nc/R groups (*p* < 0.05, Fig. 5C). NPHS2 overexpression was accompanied by enhanced oxidative stress in both parental and resistant ccRCC cells. Through mitochondrial staining, we also observed that the proportion of JC-1 green fluorescence in the NPHS2-ov and NPHS2-ov/R groups increased significantly (*p* < 0.05), suggesting obvious mitochondrial damage (Fig. 5C). In terms of the alteration of the cellular TME, the fluorescence intensity of CD8 on T cells in the ccRCC-T cell co-culture system increased in the NPHS2-ov and NPHS2-ov/R groups, while the fluorescence intensities of PD-1 and PD-L1 on ccRCC cells decreased (*p* < 0.05), indicating that NPHS2 overexpression may reduce immune-evasive features in 786-O, A498, 786-O/R, and A498/R cells (Fig. 5D). Notably, by flow cytometry, we also found that CD69^+^ and IFN-γ^+^ were increased, while PD-1^+^ and TIM-3^+^ were decreased in the NPHS2-OV and NPHS2-OV/R groups (*p* < 0.05, Fig. 5E), confirming that overexpression of NPHS2 could enhance T cell activation. And inhibit the exhaustion of T cells.

### 3.6 Effect of NPHS2 on ccRCC Living Tumors

**Figure 6:**
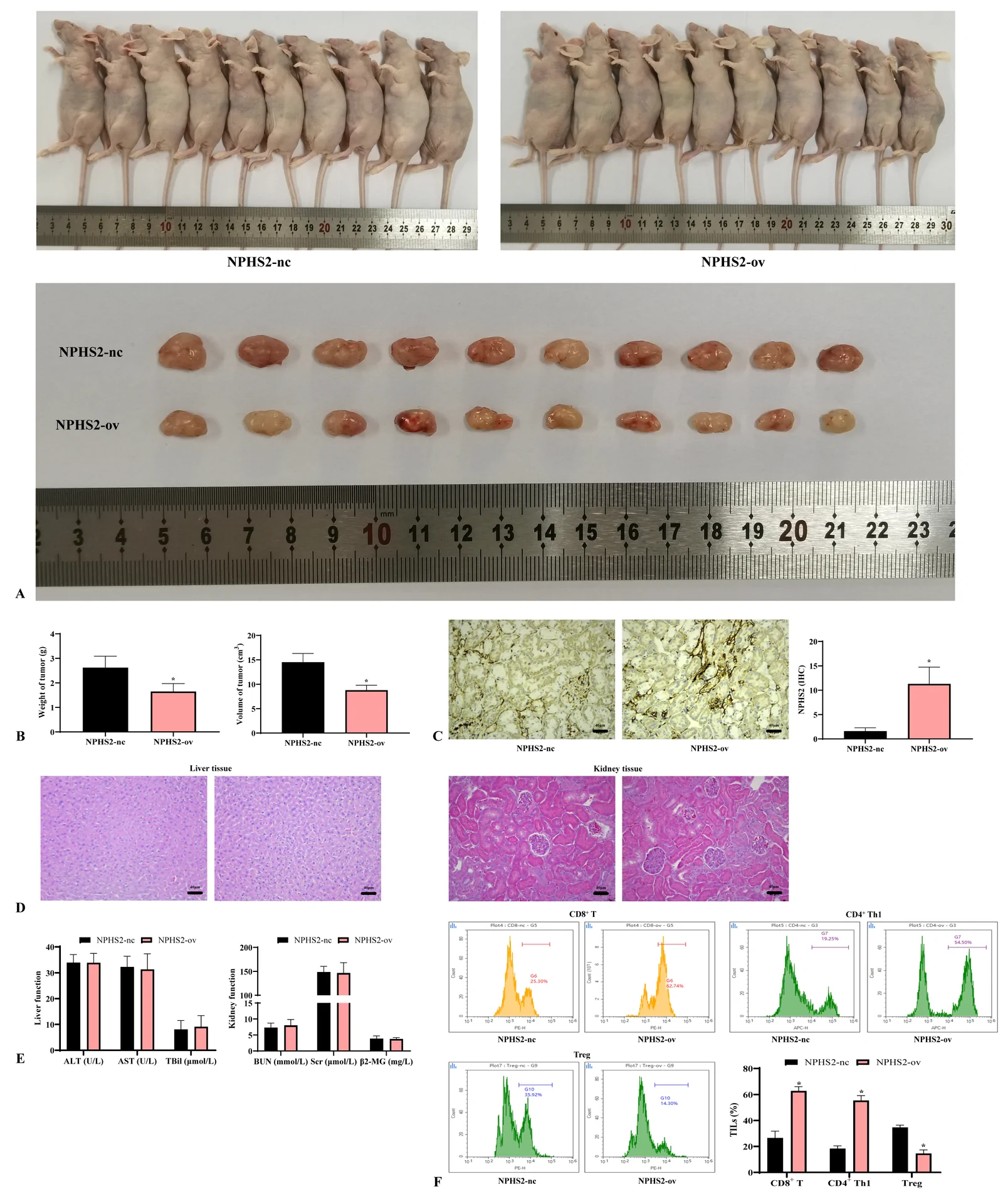
**Effect of NPHS2 on ccRCC living tumors.** (**A**) Photographs of tumor *in vivo* and tumor *ex vivo* (*n* = 10). (**B**) Weight and volume of the tumor (*n* = 10). (**C**) IHC detection of NPHS2 expression in tumor tissues (*n* = 10). (**D**) HE staining of liver and kidney tissues (*n* = 10). (**E**) Results of biochemical tests of liver and kidney function (*n* = 10). (**F**) TILs were detected by flow cytometry. vs. NPHS2-nc group *p < 0.05.

To assess the *in vivo* effect of NPHS2, we established a 786-O xenograft model and examined tumor growth after NPHS2 overexpression. After cultivating tumor-bearing mice, it was found that the tumor volume and weight of the NPHS2-ov group were significantly smaller compared to the NPHS2-nc group (*p* < 0.05, Fig. 6A,B). According to IHC staining, the NPHS2-ov group had an obviously higher NPHS2-positive rate than the NPHS2-nc group (*p* < 0.05, Fig. 6C), indicating that increasing the expression of NPHS2 has an inhibitory effect on ccRCC tumor growth. Meanwhile, the liver and kidney function test results in mice showed no significant difference between the NPHS2-ov group and the NPHS2-nc group (*p* > 0.05, Fig. 6D, E), and tissue HE staining indicated no obvious pathological changes (Fig. 6D, E), confirming that increasing NPHS2 expression through lentiviral vectors has no significant toxic side effects. We detected the composition of TILs in the two groups of mice, and the results showed that the proportion of CD8^+^ T cells and CD4^+^ Th1 cells in the TILs (derived from nude mouse innate immune cells and tumor-recruited immune cells) of the NPHS2-OV group was higher than that in the NPHS2-NC group (*p* < 0.05), suggesting that the overexpression of NPHS2 could promote the infiltration of TILs (Fig. 6F). It also improved the progression of ccRCC by virtue of its immunomodulatory effects.

## 4 Discussion

Clinically, a better understanding of the pathogenesis of ccRCC is considered to be the key to finding new diagnostic and therapeutic options [38]. Notably, the pro-tumor effects of NPHS2 silencing on immune escape and sunitinib resistance are indirect effects mediated by the downregulation of NPHS2 expression, rather than a direct causal effect of silencing itself. In the present study, NPHS2 emerged from public dataset analysis as a candidate gene of interest in ccRCC. Its epigenetic modification ability has had a remarkable impact on the biological behaviors and drug resistance of ccRCC cells, fully demonstrating the application value of NPHS2 in the diagnosis and treatment of ccRCC in the future.

First, we found a total of 159 DEGs (20 upregulated and 139 downregulated) from the GSE68417 dataset, all of which are of important clinical significance. After mapping the PPI network of these DEGs, it was found that SL34A1 was at the core of the network. In the study by Qiu et al., they have confirmed that SLC34A1 is related to the poor prognosis of ccRCC [39]. However, as mentioned above, in the research of modern malignant tumor diseases, the crucial point that we cannot overlook is the change in its epigenetic modification behavior, the abnormality of which has been confirmed to lead to the occurrence of tumors [40]. In this regard, we conducted functional enrichment analysis on these DEGs and found that multiple of them were involved in abnormal changes in cell morphology and function. What we found was, in GO analysis, NPHS2 was enriched in kidney development (urogenital system development, renal system development, renal system development, renal system development, renal system development). Metanephric glomerular epithelium development/differentiation and cell connection/cytoskeleton organization, cell junction, actin cytoskeleton organization, and cytoskeleton organization. These processes are critical in the development and progression of cancers, including ccRCC, and are known to be extensively affected by epigenetic regulation. At the same time, NPHS2 was enriched in the plasma membrane part (plasma membrane part and membrane microdomain) and extracellular exosome/vesicle, which may be related to their functions and potential epigenetic downstream effects. This, combined with its observed downregulation in ccRCC databases, prompted us to investigate the potential role and regulatory mechanism of NPHS2 in ccRCC. Therefore, we examined NPHS2 protein expression in ccRCC tissues and adjacent non-tumor tissues and found that NPHS2 was significantly downregulated in cancer tissues, suggesting that NPHS2 is involved in the initiation and progression of ccRCC. However, the relationship between NPHS2 and epigenetic modifications in ccRCC remains to be verified.

Epigenetic modifications are present throughout the entire process of the occurrence and development of various malignant tumors [41]. Abnormal methylation in the promoter region, as one of the most common epigenetic regulatory mechanisms, is widely involved in the regulation of various gene expressions in cancer cells [42]. DNA methylation mostly occurs on CpG islands in the gene promoter region. Under the catalysis of methyltransferase, the active methyl group is transferred from S-adenosylmethionine to the fifth position of cytosine to form 5-methylcytosine. Methylation in the gene promoter region can lead to transcriptional repression [43,44]. By analyzing changes in NPHS2 expression in 786-O cells treated with the DNA methylation inhibitor 5′-Aza-CdR, it was found that 5′-Aza-CdR significantly increased NPHS2 protein expression in 786-O, suggesting that there may be highly methylated modifications in the NPHS2 gene promoter region in ccRCC cells. Through MSP detection of cancer tissues and paired cancer tissue samples, it was further confirmed that there was a high methylation phenomenon in the promoter region of the NPHS2 gene in ccRCC tissues [45], while its clinical expression was consistently downregulated, consistent with the results of GEO2R mentioned above. These findings support a link between promoter hypermethylation and reduced NPHS2 expression in ccRCC, suggesting that epigenetic silencing may underlie its loss in tumor tissue [46].

In this regard, we designed a lentiviral vector that up-regulated NPHS2 expression and observed the changes in the viability and biological behavior of ccRCC cells after transfection. After upregulating NPHS2, the activity of ccRCC was significantly reduced, the malignant invasion and migration abilities of cells were effectively inhibited, and the cells were largely blocked in the G0/G1 phase, confirming that upregulating the expression of NPHS2 is conducive to inhibiting the malignant growth of ccRCC. Similarly, Xiong et al. showed that luteolin ameliorates hyperglycemia-induced nephrotoxicity by upregulating NPHS2 expression [47], which supports our findings. In terms of biological behavior, we also found that compared with the NPHS2-nc group, the EMT of the NPHS2-ov group was significantly inhibited, while the cellular oxidative stress damage was aggravated and the mitochondrial damage was activated, suggesting that NPHS2 is related to multiple biological behaviors in the progression of ccRCC. Among them, EMT and cellular oxidative stress damage are not only important pathological links of tumor cells but also typical manifestations under the influence of the TME. For example, EMT promotes immune escape by inducing CD70 in non-small cell lung cancer [48], and SPOCK1, as a potential prognostic and therapeutic biomarker for lung adenocarcinoma, is related to EMT and immune escape [49]. In a urine mass spectrometry analysis of COVID-19 patients by Chavan et al., it was also mentioned that the enrichment of NPHS2 in immune responses is an important factor in the pathogenesis of the disease [50]. Similarly, for ccRCC, the change in the TME is also a crucial aspect that cannot be overlooked [51]. Studies have confirmed that the TME not only participates in the occurrence and development of tumors but also is the key to drug resistance in ccRCC cells [52,53]. To further explore the immune context of NPHS2, we examined its association with immune infiltration patterns in public databases. This opposing prognostic trend in KIRP is likely attributable to the profound molecular and pathological heterogeneity across different renal cell carcinoma subtypes [54]. Unlike ccRCC, which is characterized by VHL gene inactivation and prominent cytoplasmic lipid accumulation, KIRP is driven by distinct oncogenic pathways (e.g., MET, FGFR2 mutations) and harbors a unique tumor immune microenvironment composition. Additionally, NPHS2 may exert divergent regulatory effects in different renal epithelial cell lineages, leading to its contrasting biological functions in ccRCC and KIRP. Further subtype-specific studies are warranted to elucidate the precise regulatory mechanisms of NPHS2 in renal cell carcinoma. The discrepancies between the database immune infiltration results and our *in vitro*/*vivo* experimental findings may be attributed to three main factors: first, the high sample heterogeneity in public databases, which includes patients with different clinical stages, treatment histories and genetic backgrounds; second, the *in vitro* co-culture system and animal model cannot fully recapitulate the complex tumor immune microenvironment of human ccRCC; third, the immune infiltration analysis in databases is based on bulk RNA-seq data with limited resolution, while our experimental detection is based on direct cellular staining with higher single-cell level specificity. The results showed that NPHS2 was not significantly correlated with the infiltration of immune cells in various kidney tumors including ccRCC. Only in KIRP did we find that when accompanied by immune infiltration of B cells and CD8^+^ cells, the prognostic survival time of patients with high expression of NPHS2 was reduced. This is inconsistent with the above GEO2R and clinical detection results, which we speculate may be related to the relatively small amount of NPHS2-related data recorded in the database. In this regard, we need to conduct follow-up investigations of clinical pathology for verification. However, under different copy number variations, significant changes were found in all immune cells of ccRCC, which fully indicates the important potential impact of interfering with NPHS2 expression on the TME of ccRCC. Through fluorescence staining, we found that the fluorescence intensity of CD8 increased in the NPHS2-ov group, while PD-1 and PD-L1 decreased, which preliminarily corroborates this view. This observation suggests that NPHS2 may be relevant to treatment response and could merit further evaluation in the context of resistance to targeted therapy and immunotherapy [55].

To verify the above inference, we conducted further analysis from two perspectives. First, we constructed a sunitinib-resistant cell line of ccRCC by the gradient method, which is also the most commonly used method for constructing ccRCC-resistant cell lines in clinical research [56]. Compared with conventional 786-O and A498, 786-O/R and A498/R showed a significant increase in cell activity under the same concentration of sunitinib intervention, confirming their drug resistance [57]. Subsequently, after using the lentiviral vector that increased NPHS2 expression, we obtained experimental results with the same trend as 786-O and A498 above, that is, after up-regulating NPHS2 expression, the activity of 786-O/R and A498/R decreased, invasion and migration were inhibited, and apoptosis increased. Furthermore, the progression of EMT in 786-O/R and a498/R was effectively controlled, and the cell oxidative stress damage was activated, suggesting that the drug resistance of 786-O/R and a498/R is significantly reversed, which is speculated to be related to changes in the cellular TME regulated by NPHS2 [58]. The fluorescence staining results confirmed that the CD8 fluorescence intensity was elevated, and the results of PD-1 and PD-L1 were also consistent with our inference [59]. Moreover, overexpression of NPHS2 reactivated the early markers of T cell activation (CD69^+^) and effector function markers (IFN-γ^+^), while decreasing the expression of PD-1^+^ and TIM-3^+^ in both normal ccRCC cells and resistant ccRCC cells [60]. Together, these data suggest that NPHS2 overexpression is accompanied by a more active T-cell state in ccRCC [61]. From a translational perspective, restoration of NPHS2 expression may deserve further study as a strategy to improve therapeutic sensitivity in ccRCC [62]. Based on our KEGG enrichment finding that DEGs were significantly enriched in the MAPK signaling pathway, we hypothesize that NPHS2 modulates EMT, oxidative stress, immune checkpoint expression and sunitinib resistance in ccRCC cells by regulating the MAPK signaling pathway [63]. Evidence from TKI-resistant ccRCC models indicates that activation of MAPK-related signaling pathways, including the TRAF6/MAPK/AP-1 axis, contributes to acquired resistance and aggressive biological behavior [64]. Therefore, hypermethylation-driven NPHS2 silencing may promote EMT progression, immune-evasive features, and sunitinib resistance partly through MAPK-related signaling, although this hypothesis requires further pathway-specific validation. In contrast, NPHS2 upregulation may inhibit the MAPK signaling pathway to reverse these malignant biological behaviors. Subsequent studies will validate this hypothesis through pathway activation and inhibition assays. Of course, there are still many issues to be addressed for its clinical application. The first is the precise impact of the targeted elevation of NPHS2 in live animals. To clarify, we constructed a ccRCC tumor-bearing mouse model and conducted preliminary verification by injecting a lentivirus solution with interfered expression of NPHS2. The tumor growth in the NPHS2-ov group of mice was significantly inhibited, once again validating the inhibitory effect on tumor activity after increasing NPHS2 expression. Besides, no obvious pathological changes were observed in the liver and kidney functions and tissue section examinations of the mice. Finally, we also found that achieve a tumor suppressive effect, which again highlighted the immunomodulatory role of NPHS2 in ccRCC [65]. In this xenograft model, NPHS2 overexpression did not produce overt hepatic or renal toxicity, although its safety profile requires further evaluation. However, there are still differences between animal samples and humans. To fully understand its clinical application value, more experimental verification is required.

This study should be interpreted in light of several limitations. For example, the results of immune infiltration and prognosis analysis (especially the poor prognosis of high NPHS2 expression in KIRP) also need more clinical data from more independent cohorts or larger sample sizes to verify their generalization and clinical significance. Second, although studies have observed that upregulation of NPHS2 inhibits EMT, promotes oxidative stress damage, mitochondrial damage, affects immune checkpoint molecule expression, and reverses drug resistance. However, how NPHS2 specifically regulates these downstream signaling pathways and effector molecules still needs further investigation. Additionally, this study only utilized two ccRCC cell lines (786-O and A498), which may limit the generalizability of our findings. The immunodeficient nude mice used in *in vivo* experiments cannot fully recapitulate the complete and complex tumor immune microenvironment of humans, precluding an accurate reflection of the interaction between NPHS2 and the host immune system. In addition, the *in vivo* experiment lacked a sunitinib positive control group; subsequent studies will add this group to evaluate NPHS2’s clinical translational value in reversing drug resistance. The extremely small clinical sample size (*n* = 8) only allows for exploratory analysis of NPHS2 expression and methylation status, and the generalizability of the results is limited, which is the most important limitation of this study. In subsequent studies, we will expand the clinical sample size for further validation and utilize the TCGA-KIRC public dataset to verify the correlation between NPHS2 methylation and expression in ccRCC [66], as well as perform survival analyses and clinicopathological correlation analyses to enhance the robustness of our conclusions. The bioinformatic analysis in this study was based only on the GSE68417 dataset; subsequent studies will incorporate additional public datasets, including TCGA-KIRC and other GEO cohorts [67], conduct multivariate survival analysis, and systematically evaluate NPHS2 methylation data from TCGA to further improve the comprehensiveness and depth of the bioinformatic analysis. The immune microenvironment analysis in this study lacks more in-depth quantitative stratification and subgroup analysis; subsequent studies will combine single-cell RNA-seq data to clarify the specific regulatory relationship between NPHS2 and immune cell subsets in ccRCC [68].

## 5 Conclusion

NPHS2 was downregulated in ccRCC and showed an association with promoter hypermethylation in an expanded paired clinical cohort. In cellular and xenograft models, restoration of NPHS2 expression was associated with suppression of malignant phenotypes, modulation of immune-associated features, and attenuation of resistant characteristics in sunitinib-resistant ccRCC cells. These findings support NPHS2 as a candidate molecule of interest in ccRCC and justify further multicohort validation and mechanistic investigation.

## Data Availability

The data that support the findings of this study are available from the corresponding author upon reasonable request.
